# Evaluating Feasibility and Acceptability of the “My HeartHELP” Mobile App for Promoting Heart-Healthy Lifestyle Behaviors: Mixed Methods Study

**DOI:** 10.2196/66108

**Published:** 2025-05-02

**Authors:** Jina Choo, Songwhi Noh, Yura Shin

**Affiliations:** 1 College of Nursing, Korea University, 145, Anam-ro, Seongbuk-gu, Seoul, 02841, Republic of Korea, 82 2-3290-4925; 2 Transdisciplinary Major in Learning Health Systems, Department of Health Sciences, Korea University Graduate School, Korea University, 145, Anam-ro, Seongbuk-gu, Seoul, 02841, Republic of Korea

**Keywords:** healthy lifestyle, primary prevention, cardiovascular diseases, community health nursing, mobile app

## Abstract

**Background:**

Few mobile apps have strategies for self-monitoring multiple heart-healthy behaviors simultaneously, as well as automated and tailored feedback on individual behavioral outcomes for cardiovascular health. An app named “My HeartHELP” was developed for the general adult population to promote 6 heart-healthy lifestyle behaviors—physical activity, nonsedentary behaviors, healthy eating behaviors, nonsmoking, no alcohol binge drinking, and self-assessment of body weight. Three behavioral strategies were used: (1) text messaging the users for information on cardiovascular health, (2) self-monitoring of 6 heart-healthy behaviors to fill out the blanks of behavioral items, and (3) automated and tailored feedback messaging to users for behavioral outcomes obtained from self-monitoring.

**Objectives:**

This study aimed to evaluate the feasibility and acceptability of the “My HeartHELP” app.

**Methods:**

The participants were 29 community residents in Seoul, South Korea, who met at least 1 criterion of metabolic syndrome. To evaluate the feasibility, we assessed 3 records, which are as follows: First, the “record for self-monitoring” was determined as feasible if an average percentage for each of the 6 behaviors over 4 weeks was 75% or higher based on percentages of participants who completed to record each of 6 heart-healthy behaviors. Second, the “record for access to the app” was determined as feasible if users accessed at least once a day on average per week. Third, “records for behavioral changes” over 4 weeks were collected via a self-reported questionnaire. To evaluate acceptability, we used an assessment tool comprising 12 items that included subscales for comprehensibility, ease, health benefits, technical completeness, overall satisfaction, and recommendation to others on a 5-point Likert scale. Acceptability was determined as acceptable if the average scores for the total scale and each subscale were 3.5 points or greater. Second, qualitative data were collected through 2 focus groups, each consisting of 14 or 15 participants. All data were collected in June and July 2022.

**Results:**

During the 4 weeks, 95.6% (range: 85.8%-97.4%) of the participants adhered to more than 75% of “completion of daily self-monitoring of each heart-healthy behavior,” having met the criterion. The participants accessed the app on average 1.8 (SD 1.70) times per day, meeting the criteria. Participants had positive behavioral changes in all 6 behaviors, of which nonsedentary behavior (10%-28%; *χ*^2^_1_=1.76; *P*<.001) and non–fast-food intake were especially statistically significant (72%-93%; *χ*^2^_1_=5.64; *P*=.03) over 4 weeks. Participants reported 3.8 points for a total score of acceptability and more than 3.5 points for all subscales, which met the criterion. Qualitative data obtained from focus groups indicated that automated and tailored feedback messages motivated participants to promote healthy lifestyles.

**Conclusions:**

The “My HeartHELP” app may be a feasible and acceptable mobile app to promote self-monitoring and possibly behavioral changes in heart-healthy lifestyle behaviors.

## Introduction

Cardiovascular disease is preventable through the promotion of heart-healthy behaviors, such as physical activity, nonsmoking, nonsedentary, and healthy eating behaviors, refraining from alcohol binge drinking, and self-assessment of body weight [1,2]. Each additional behavioral modification may reduce the risk of cardiovascular disease [1,3]. However, only a small percentage of the population is known to adhere to these behaviors [4,5]. In this context, strategies are needed to effectively change multiple heart-healthy behaviors that are accessible to everyone, even before the onset of heart disease, and in community settings.

An app of mobile health technologies that allow community-dwelling individuals to monitor their own health behaviors and promote health behaviors in their daily lives may be needed to effectively change heart-healthy behaviors [6]. Mobile apps have the advantages of self-directedness, instant connectivity, ubiquity, personalization, and interactive learning through social engagement [7], which may enhance self-monitoring and practice of health behaviors without time and space constraints. However, few mobile apps are equipped with strategies for a 1-stop approach to monitor multiple heart-healthy behaviors that may correlate with each other [8] and target individuals without cardiovascular disease in a community setting. However, evidence indicating that mobile health technologies can effectively improve heart-healthy behaviors is lacking. This may be due to a lack of empathy and ineffective educational effects through active and persistent interactions with cardiovascular experts [9]. In this context, mobile technologies should be designed to enhance the self-monitoring of their own behaviors and interactions between mobile app users and experts in the context of cardiovascular prevention and promotion.

Several mobile apps have been developed to monitor health behavior changes [10,11] in the context of disease management for hypertension, diabetes, cardiovascular disease, cancer, and obesity [12-15]. However, the monitoring strategies used in each study were inconsistent [16]. In addition, the design of such mobile apps for health behavioral interventions has been confined to the process of self-reporting individual health behaviors in the form of a checklist [17]. Finally, few health-promoting mobile apps have automated feedback text messages for individual behavioral outcomes achieved with the self-monitoring of multiple lifestyle behaviors in the context of cardiovascular health.

The Information-Motivation-Behavioral Skills model (IMB model) is widely used as a theoretical framework for predicting health behavior changes [18]. The IMB model addresses the core constructs of information, motivation, and behavioral skills as prerequisites for health behavior change and assumes that when an individual acquires sufficient information related to health behavior, is motivated to change behavior, and improves behavioral skills, their behavior change and maintenance are promoted, which in turn can improve health levels [18]. In this theoretical background, we designed a mobile app to be embedded with behavioral strategies to encourage individuals to be informed, motivated, and skilled in behavior change by using cognitive-behavioral strategies addressed by the American Heart Association [19].

One such app is the “My HeartHELP” app, which was developed for the general adult population to promote heart-healthy lifestyle behaviors based on the constructs of information, motivation, and behavioral skill in the IMB model [20]. The “My HeartHELP” app is an abbreviation derived from the Heart-Healthy Lifestyle-Promoting app [21]. This app targets 6 heart-healthy lifestyle behaviors, such as physical activity, nonsedentary behaviors, healthy eating behaviors, nonsmoking, refraining from alcohol binge drinking, and self-assessment of body weight [22,23]. Of the 6 behaviors, 5 were selected based on the rationale that the US Centers for Disease Control and Prevention emphasizes key preventive behaviors for heart health, including healthy food and beverage choices, maintaining a healthy weight, engaging in regular physical activity, and nonsmoking [23]. An additional behavior, nonsedentary behavior, was incorporated due to the established association between sedentary behavior and increased cardiovascular risk [1]. The “My HeartHELP” app was designed with three behavioral strategies : (1) text messaging users for information on cardiovascular health, (2) self-monitoring of 6 lifestyles, and (3) automated and tailored feedback text messaging to users for behavioral outcomes obtained from self-monitoring. To increase the construct of information in the IMB model, texts on knowledge about cardiovascular prevention [24] was provided via text messaging. To increase the constructs of motivation and behavioral skills in the IMB model, goal setting [25,26], self-reflection via self-monitoring of behaviors [27], and positive reinforcement and tailored real-time feedback of behavioral performance via text messages [28,29] were installed in the “My HeartHELP” app. In particular, providing feedback is a well-established strategy in behavioral interventions. A Cochrane review underscored its importance in facilitating behavior change, particularly in contexts where substantial improvement is needed [30]. This aligns with the American Heart Association’s cognitive-behavioral strategies, which also emphasize feedback as a key component in sustaining behavioral improvements [19].

In this context, we aimed to assess the feasibility and acceptability of the “My HeartHELP” app in this study to conduct a future randomized controlled trial (ISRCTN83643383).

## Methods

### Study Design and Sample

This study used a mixed methods design [31] with a single-group approach to evaluate the feasibility and acceptability of the “My HeartHELP” app using both quantitative and qualitative data. This study design was chosen to incorporate qualitative data, in addition to quantitative data, to facilitate a more comprehensive evaluation of app users’ perceptions of its acceptability, including its health benefits and overall usefulness. The app was implemented over a 4-week period. Data were collected at both pretest and posttest stages. At each stage, a questionnaire was administered, and at the posttest stage, focus group discussions were conducted.

The participants were 29 adults. They were recruited by the following ways: First, we posted a recruitment notice on the bulletin boards of the university affiliated with the researchers. Second, we additionally posted it on a community-based mobile commercial and life platform (ie, Karrot app). In this way, the Karrot automatically displays the notice to residents in 174 neighborhoods across 5 municipal districts surrounding the university in Seoul, South Korea. Individuals interested in participating in the study contacted the researchers via mobile communication platforms (eg, the KakaoTalk app) or by phone, after which an eligibility screening was conducted based on the following criteria. The inclusion criteria were adults (1) aged between 20 and 64 years who had at least one of the metabolic syndrome components (ie, abdominal obesity, hypertension, dyslipidemia, or glucose intolerance). The exclusion criteria were individuals diagnosed with (1) cardiovascular disease and undergoing treatment, (2) major depression or anxiety disorder, (3) physical impairment, (4) cognitive impairment, and (5) difficulties in using a mobile app.

The sample size was determined to be 30 adults based on a study by Symer et al [32] that tested the validity of a mobile app. Of the 30 participants, 1 lost contact during the study period of 4 weeks and 29 were included in the final analysis of quantitative and qualitative data.

### Overview of the “My HeartHELP” App

**Figure 1.**
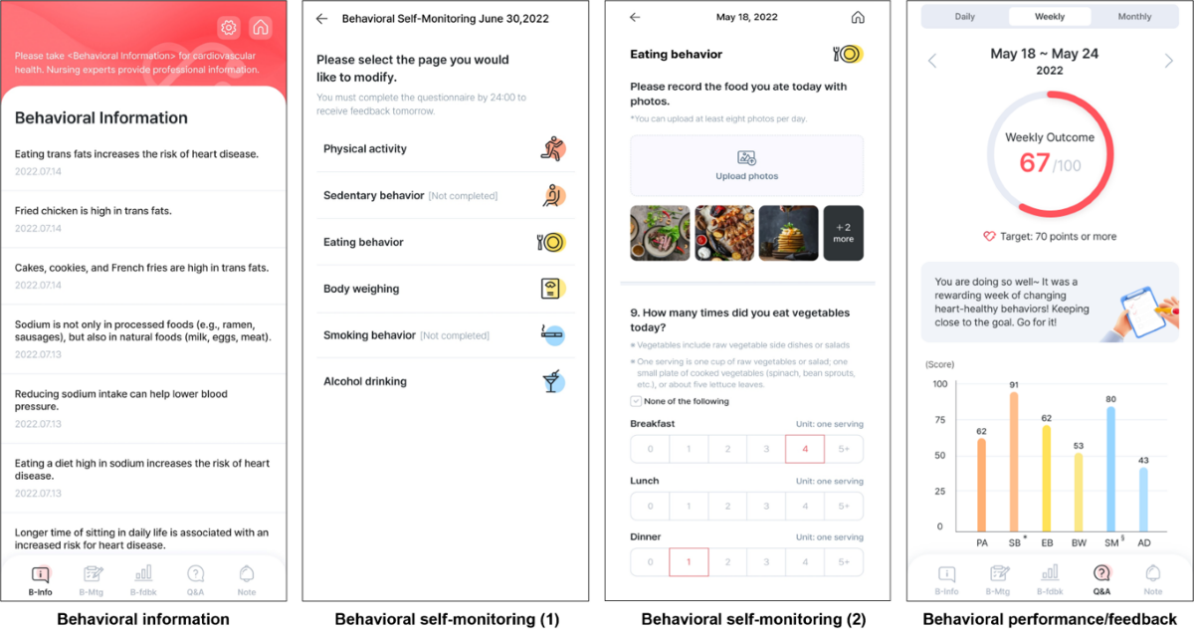
Overview of the “My HeartHELP” app. This mobile app was developed in Korean and designed based on 3 behavioral strategies: behavioral information, behavioral self-monitoring, and behavioral performance/feedback. In this study, the app was applied over a 4-week period to 29 participants residing in Seoul, South Korea, who were at risk for cardiovascular disease. Pre- and posttests were performed to evaluate the feasibility and acceptability of the app.

The “My HeartHELP” app was designed with 3 behavioral strategies, that is, behavioral information, behavioral self-monitoring, and behavioral performance/feedback. The structure and content of the app are described in detail in Figure 1 and Table 1.

**Table 1.** The structure of the “My HeartHELP” app: its strategies and contents.

Strategies	Contents	
Behavioral information	Behavioral knowledge A total of 43 text messages for symptoms and characteristics of cardiovascular disease, cardiovascular risk factors, and heart-healthy behaviors 8‐11 texts delivered per week to participants From Monday to Friday at 1:00 PM over 4 weeks	
Behavioral self-monitoring	Ensuring that the participants recorded the extent to which they practiced each of the 6 heart-healthy behaviors during the entire day Physical activity (steps of walking and types and minutes of moderate-intensity exercise) Sedentary behavior (time, hours) Eating behavior (fruit and vegetable, sugar-sweetened beverage, fast foods, foods high in saturated fat or trans fat, whole grain, and sodium intake) Self-measurement of body weight (kg per day) Smoking behavior (cigarettes per day) Alcohol-drinking behavior (drinks per day)	
Behavioral performance/feedback	Interface displays according to the level of heart-healthy behavior achieved over the entire day, 7 days (a week), and 30 days (a month) Based on the algorithm for behavioral target goals that the authors had installed for each of 6 heart-healthy behaviors Automated and tailored feedback text messaging Daily at 9:30 AM from Monday to Friday Weekly at 9:30 AM on Monday Monthly at 9:30 AM on the first Tuesday Example: “You did a good job today” or “You almost reached your goal today. Just a little more effort, and you will reach your goal. Cheer up!”	

#### Behavioral Information

“Behavioral information” refers to text messaging of heart-health–related information on the characteristics of cardiovascular diseases and heart-health–related behaviors (Figure 1 and Table 1). Text messages were delivered as push notifications 5 times per week at 1:00 PM over a course of 4 weeks.

#### Behavioral Self-Monitoring

“Behavioral self-monitoring” refers to the participants’ recording the extent to which they achieved 6 heart-healthy behaviors (Figure 1 and Table 1). Physical activity was recorded into 2 types: daily step counts for walking and daily time duration for moderate-intensity aerobic exercise; sedentary behavior recorded screen time (hours); and eating behavior recorded frequencies and types of whole grain intake, fruit and vegetable intake, sugar-sweetened beverage intake, sodium intake, fast-food intake, and intake of foods high in saturated fat. The participants were instructed to weigh body weights to measure and record. Participants were asked to daily record whether they smoked cigarettes and, if so, how many cigarettes they smoked. The participants were requested to record the frequency of alcohol consumption with a standard glass [33,34].

#### Behavioral Performance/Feedback

“Behavioral performance/feedback” refers to automatized and tailored interface displays including diagram and texts (Figure 1 and Table 1) according to outcomes adhered to 6 heart-healthy behaviors achieved over 1 day, 7 days (a week), and 30 days (a month), based on the algorithm for behavioral target goals that the authors had installed for the 6 heart-healthy behaviors.

The frequency of feedback delivery was structured as daily, weekly, and monthly, based on the rationale that frequent feedback may enhance motivation, self-monitoring, and goal attainment for behavioral change [35], and that daily, weekly, and monthly self-monitoring may support positive health outcomes [35-37]. Push notifications were delivered to request access to the interface display.

Daily outcomes are displayed as achieved scores out of 10 points (eg, 6/10) (Figure 1), weekly outcomes as achieved scores out of 100 points and bar graphs per behavior (Figure 1), and monthly outcomes as achieved scores out of 100 points and the symbol of the heart if achieved. Specifically, the daily outcomes were obtained by achieved scores from the components of 6 behaviors. Physical activity was evaluated by awarding 1 point for achieving a minimum of 8000 steps per day [38] or engaging in at least 30 minutes of moderate-intensity exercise per day [39]. Nonsedentary behavior was assessed by awarding 1 point for limiting sedentary time to less than 2 hours per day [40]. Eating behaviors were evaluated across multiple dietary components. Fruit and vegetable intake was assessed by awarding 1 point for consuming a minimum of 5 servings per day [41]. Similarly, 1 point was awarded for abstaining from sugar-sweetened beverages [42], avoiding fast-food consumption [43], refraining from consuming foods high in trans fats [44], and maintaining sodium intake below 1500 mg per day [45]. Self-monitoring of body weight was assessed by awarding 1 point for weighing oneself daily [46,47]. Smoking behavior was evaluated by awarding 1 point for complete abstinence from smoking [48]. Finally, alcohol consumption was assessed by awarding 1 point for limiting intake to fewer than 5 drinks per day for men and fewer than 4 drinks per day for women [49].

Weekly outcomes were derived from the average scores obtained over 7 days, which were then converted to a 100-point scale and categorized into 3 ranges: ≥70, 50‐69, and <50. Feedback texts were generated based on these score ranges. Similarly, a monthly outcome was calculated from the average scores over the previous 4 weeks, converted to a 100-point scale, and classified into 2 categories: ≥70 and <70. Corresponding feedback texts were generated accordingly.

To account for users’ baseline characteristics and minimize redundant data entry, participants who self-identified as nonsmokers and nondrinkers were provided with a tailored interface that excluded smoking- and alcohol-related inputs. In addition, they received automated feedback reinforcing their adherence to these behaviors.

### Study Procedure and Data Collection

The study procedure and data collection were conducted in 3 sequential stages between June and July 2022 in Seoul, South Korea. Specifically, the pretest was conducted on June 16‐17 (stage 1), followed by the 4-week implementation of the “My HeartHELP” app from June 20 to July 17 (stage 2). The posttest and focus group sessions took place on July 21‐22 (stage 3).

Stage 1: The pretest took place on the same day using a self-reported questionnaire on general characteristics and heart-healthy behaviors. After the pretest, the pre-education session for informing the usage of the “My HeartHELP” app was conducted as a face-to-face group session. At this session, the principal investigator (JC) presented a lecture to inform the characteristics of heart disease and the importance of promoting heart-healthy behaviors for 20 minutes. This lecture aimed to provide background knowledge on the variables measured by the mobile app and to facilitate understanding of the app’s usage. Following the lecture, the researchers provided a detailed explanation of the structure and usage of the “My HeartHELP” app, along with an overview of the overall research process. Stage 2: The implementation of the “My HeartHELP” app took place for 4 weeks without contact with the researchers in between. Stage 3: Postsurvey and focus group session took place on the same day after the end of the 4-week implementation. The postsurvey session was administered using the self-reported questionnaire with heart-healthy behaviors (ie, repeated measures) and the acceptability of the app. Focus group sessions, each lasting 60 minutes, were conducted with 2 groups comprising of 14‐15 participants per group to assess the acceptability of “My HeartHELP” app. The principal investigator (JC) facilitated the discussion by using open-ended questions, probing questions, and laddering techniques [50] to encourage participants to freely express their thoughts and provide in-depth feedback. Key topic areas included overall impressions, strengths and weaknesses, comprehensibility, perceived health benefits, and usability (ie, ease of use and convenience in navigation and operation). In addition, challenges such as system errors, data input difficulties, and technical issues were explored. Participants also suggested improvements related to system errors, data input difficulties, and readability concerns. All sessions were audio-recorded and transcribed verbatim for analysis.

### Study Variables and Metrics

General characteristics included sex, age, college-educated status, employment status, exposure to heart health education, history of chronic diseases (ie, hypertension, diabetes mellitus, or dyslipidemia), and self-reported height and body weight.

#### The Feasibility of “My HeartHELP”

To evaluate the feasibility of “My HeartHELP,” we assessed the following 3 behavioral records: First, the “record for self-monitoring” was determined based on the data of the percentage of participants who completed recording each of 11 behavioral components (Figure 1) of the 6 heart-healthy behaviors per day, and their average percentage over 4 weeks was assessed. We determined that this was feasible if the average percentage was 75% or higher [32]. Second, the “record for access to the app” was determined based on the data of a mean of daily access counts per week. Next, we determined whether it was feasible for the users to access the app at least once a day on average per week. Third, the “record for behavioral changes” was determined as feasible if participants showed improvements in each of the 6 behaviors when comparing pretest and posttest. Among the 6 behaviors, the following behaviors were included in the analysis : Physical activity (2 items) was assessed as moderate-intensity aerobic exercise for 150 minutes per week [39]. Walking (2 item) was also assessed for 150 minutes per week [38]. Nonsedentary behavior (1 item) was assessed daily, defined as having less than 2 hours of screen time (screen time included activities such as watching TV and using a mobile phone, computer, or tablet, excluding work- or study-related activities) [40]. The intake of fruits and vegetables (1 item) was assessed as 2 or more servings per day [41]. Non—sugar-sweetened beverage intake (1 item) was assessed none per day [42]. Non—fast-food intake (1 item) was assessed as consumption less than 2 times per week [43]. Whole grain intake (1 item) was assessed as consuming 2 or more servings per day, where 1 serving equals 1 slice of whole grain bread [51]. High-risk alcohol consumption (2 items) was defined as 5 or more drinks per session for men (including 4 or more drinks per session for women) [49]. Maintaining healthy body weight (1 item) was assessed by having participants report their current weight.

#### The Acceptability of “My HeartHELP”

To evaluate the acceptability of “My HeartHELP,” we used quantitative data from an assessment tool and qualitative data from focus groups. First, we developed the assessment tool with 12 items that consisted of the following subscales: comprehensibility (2 items), ease (2 items), health benefits (5 items), technical completeness (1 item), overall satisfaction (1 item), and recommendation to others (1 item). Health benefits were categorized into “behavioral information,” “behavioral monitoring,” and “behavioral performance/feedback.” The items were scored on a 5-point Likert scale (1=not at all, 2=quite not, 3=somewhat, and 4=very much). The subscales were considered acceptable if the mean score for each subscale was 3.5 points or greater. Second, qualitative data obtained from focus groups were used. The following questions [52] were addressed: “What is your overall impression of the My HeartHELP?” “Please tell us three pros and three cons of the My HeartHELP.” “Please tell us about My HeartHELP based on (1) comprehensibility (2) health benefits (3) usability (4) challenges.” The qualitative data were considered acceptable if researchers identified meaningful quotations highlighting the benefits and usefulness of the “My HeartHELP” app in facilitating positive heart-healthy behavioral changes and improving health outcomes. While the quantitative data provided structured acceptability scores, they were limited in capturing the depth of users’ perceptions and the specific challenges encountered during app usage. To address this limitation, qualitative findings from focus group discussions were integrated, offering a more nuanced understanding of user perceptions regarding comprehensibility, perceived benefits, and challenges. This approach enabled a more comprehensive evaluation that extends beyond numerical assessments alone.

### Data Analysis

SPSS/WIN (version 28.0; SPSS, Inc) was used [53]. The significance level was set at less than .05. Participants’ baseline measures (ie, general characteristics and heart-healthy behaviors) were analyzed using descriptive statistics (frequencies, percentages, means, and SDs). To compare the changes in heart-healthy behaviors between pre- and postsurvey, McNemar’s chi-square test for dichotomous variables and the 2-tailed paired *t* test for continuous variables were conducted. The measures of feasibility and acceptability were analyzed using descriptive statistics for the quantitative data and content analysis for the qualitative data. Qualitative data obtained from focus groups were analyzed using Graneheim and Lundman’s qualitative content analysis [54]. The interview recordings were repeatedly reviewed, transcribed, and thoroughly examined to capture participants’ perspectives comprehensively. The transcribed data were then segmented into meaningful units, systematically coded, and organized into categories and subcategories to ensure a structured interpretation. Continuous comparisons were conducted across data points to refine themes and establish thematic classifications. The 2 sets of qualitative data were independently analyzed by 3 authors (JC, SN, and YS) and consolidated into meaningful themes and categories [55,56].

### Ethical Considerations

This study was approved by the institutional review board of Korea University (KUIRB-2022-0147-01). All participants provided written informed consent for their participation in the study, including permission for audio recording during the focus group discussions. All procedures were performed in accordance with the ethical standards of the institutional research committee and the 2013 Declaration of Helsinki. To ensure the privacy and confidentiality of participants, all data were deidentified and securely stored on encrypted servers, with access restricted to authorized members of the research team. Participants who successfully completed all stages of the study received a monetary compensation of KR ₩200,000 (US $137) [57]. Participants who completed only the pretest received a KR ₩10,000 (US $7) gift voucher. All details related to compensation were fully disclosed to participants during the informed consent process, prior to their enrollment in the study.

## Results

### Participant Characteristics

The participants (N=29) had a mean age of 41.1 (SD 13.03) years (Table 2). Of all the participants, 62% (18/29) were women; 79% (23/29) were college-educated; 66% (19/29) were employed; 45% (13/29) had a previous history of hypertension, diabetes mellitus, or dyslipidemia; and 69% (20/29) fell into the category of more than 25 kg/m^2^ of BMI. Moreover, 7% (2/29) of the participants had undergone a heart-health educational session.

**Table 2.** General characteristics of participants (N=29).

Participant characteristics		Values
Age (years), mean (SD)		41.1 (13.03)
Gender, n (%)		
Women		18 (62)
Men		11 (38)
College-educated, n (%)		
Yes		23 (79)
No		6 (21)
Employed, n (%)		
Yes		19 (66)
No		10 (34)
Having diseases^a^, n (%)		
Yes		13 (45)
No		16 (55)
BMI, kg/m^2^, n (%)		
≥25		20 (69)
<25		9 (31)
Exposed to heart-health educational sessions, n (%)		
Yes		2 (7)
No		27 (93)

a Having diseases indicate hypertension, diabetes mellitus, or dyslipidemia.

### The Feasibility of “My HeartHELP”

The feasibility for the “record of self-monitoring” was determined to be feasible. The record of self-monitoring for 11 behavioral components of the 6 heart-healthy lifestyle behaviors is shown in Multimedia Appendix 1. The 4-week percentages of participants who adhered to more than 75% of the completion of daily self-monitoring for each heart-healthy behavior are reported in Multimedia Appendix 1. The average percentage was 95.6%, ranging from a minimum of 85.8% (recording body weight) to a maximum of 97.4% (recording physical activity).

**Figure 2.**
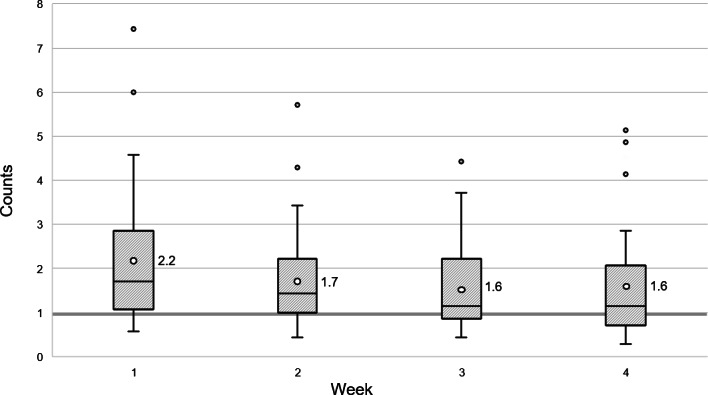
Feasibility of the “My HeartHELP” app: behavioral record of access to the app. The box plot represents the mean values of daily app access counts per week over a 4-week period based on data from 29 participants at risk for cardiovascular disease residing in Seoul, South Korea. The feasibility criterion was set at an average of at least 1 access per week.

The feasibility for “record of access to the app” was determined as feasible. Mean daily access counts per week showed 2.2 (SD 2.10) at week 1, 1.7 (SD 1.58) at week 2, 1.6 (SD 1.34) at week 3, and 1.6 (SD 1.62) at week 4. The grand mean was 1.8 (SD 1.70), which exceeded the feasibility criteria (Figure 2).

The feasibility for “record for behavioral changes” is shown in Table 3. Numbers of participants for physical activity increased from 24% (7/29) to 34% (10/29) for sufficient moderate-intensity aerobic exercise, from 59% (17/29) to 62% (18/29) for sufficient walking exercise, and from 10% (3/29) to 28% (8/29) for nonsedentary behavior (*χ*^2^_1_=1.76; *P*<.001). Regarding eating behavior, numbers of participants increased from 7% (2/29) to 21% (6/29) for intake of fruits and vegetables, from 86% (25/29) to 90% (26/29) for the intake of non–sugar-sweetened beverages, from 72% (21/29) to 93% (27/29) for the intake of non–fast foods (*χ*^2^_1_=5.64; *P*=.03), and from 62% (18/29) to 66% (19/29) for intake of whole grains. The number of high-risk alcohol drinkers decreased from 38% (11/29) to 34% (10/29). Finally, body weight changed from 76.2 kg to 76.0 kg.

**Table 3.** Changes in heart-healthy behaviors after the application of “My HeartHELP” (N=29).

Characteristics			Pretest	Posttest	Chi-square or *t* test (*df*)	*P* value
Physical activity						
Sufficient moderate-intensity aerobic exercise, n (%)					10.72 (1)^a^	.38
Yes			7 (24)	10 (34)		
No			22 (76)	19 (66)		
Sufficient walking exercise (≥150 minutes), n (%)					14.78 (1)^a^	.99
Yes			17 (59)	18 (62)		
No			12 (41)	11 (38)		
Nonsedentary behavior (<2 hours/day), n (%)					1.76 (1)^a^	<.001
Yes			3 (10)	8 (28)		
No			26 (90)	21 (72)		
Fruit and vegetable intake (≥2 servings/day), n (%)					1.13 (1)^a^	.22
Yes			2 (7)	6 (21)		
No			27 (93)	23 (79)		
Non–sugar-sweetened beverage intake (less than once per day), n (%)					7.87 (1)^a^	.99
Yes			25 (86)	26 (90)		
No			4 (14)	3 (10)		
Non–fast foods (<2 servings/week), n (%)					5.64 (1)^a^	.03
Yes			21 (72)	27 (93)		
No			8 (28)	2 (7)		
Whole grains (≥2 servings/day), n (%)					11.47 (1)^a^	.99
Yes			18 (62)	19 (66)		
No			11 (38)	10 (34)		
High-risk alcohol drinking**^b^**, n (%)					24.98 (1)^a^	.99
Yes			11 (38)	10 (34)		
No			18 (62)	19 (66)		
Body weight (kg), mean (SD)			76.2 (15.36)	76.0 (15.21)	1.22 (28)^c^	.23

a Chi-square test.

b High-risk drinking indicates ≥5 drinks per session for men (≥4 drinks per session for women).

c *t* test.

### The Acceptability of “My HeartHELP”

Acceptability using quantitative data was determined as acceptable if the scores for all the subscales of acceptability were 3.5 points (the criteria of acceptability) or greater (Table 4). The scores ranged from a minimum of 3.6 points (health benefits for information, health benefits for performance/feedback, and technical completeness) to a maximum of 4.1 points (comprehensibility).

**Table 4.** Evaluation of the acceptability for the My HeartHELP (N=29).

			Values, mean (SD)
Total score of the acceptability			3.8 (0.61)
Comprehensibility			4.1 (0.81)
Ease			3.9 (0.84)
Health benefits			
Overall			3.8 (0.87)
Behavioral information			3.6 (1.02)
Behavioral monitoring			3.8 (0.90)
Behavioral performance/feedback			3.6 (0.70)
Completeness, technical			3.6 (0.87)
Satisfaction, overall			3.8 (0.73)
Recommendation to others			3.8 (0.87)

The acceptability using the qualitative data may be determined as acceptable, as shown in Table 5. Participants mentioned that reflecting on their own heart-healthy behaviors via self-monitoring, adjusting their own heart-healthy behaviors via receiving feedback messages, and becoming motivated for weight control were health benefits. The participants mentioned that easily understood phrases were acceptable in terms of the use of easy language in the app. However, the participants suggested the following challenges for the structure and interface: differentiating between good and bad behaviors by color, including options for a variety of dietary behavior options, and installing additional reminder alarms (Table 5).

**Table 5.** Themes and quotations extracted using focus group discussion (N=29).

Themes and categories		Quotations
Comprehensibility		
Understanding phrases easily		“Overall, I think it was easy to understand. The language used was everyday language that we encounter in our daily lives, so it was not difficult.”
Benefits		
Reflecting on their own heart-healthy behaviors via self-monitoring		“I habitually consume large yogurt drinks several times a day. However, as I continued to record them under the sweetened beverage category, I realized that just two of these large yogurts exceeded my daily sugar intake limit, leading me to stop consuming them.”
		“I used to drink alcohol quite often even when dining alone, but after seeing the records I entered, I found myself not ordering alcohol even when getting delivery food.”
Adjusting their own heart-healthy behaviors via receiving feedback messages		“When I woke up each day, I first checked the daily achievement scores in ‘My HeartHELP’ After that, I started paying more attention to what I ate and definitely did more exercise throughout the day.”
Getting motivated for weight control		“I’m maintaining my 3 kg weight loss because I have been using it continuously to motivate me to lose weight.”
Challenges		
Differentiating good and bad behaviors by colors		“It would be good to distinguish unhealthy foods, like salty ones, by marking them with red or orange, and to mark healthy foods with yellow.”
Including options of a variety of dietary behavior options		“I think it would have been better if I could enter other foods not listed. Or, it would be nice if there was an explanation that allowed me to include foods not presented based on my own judgment.”
Installing additional reminder alarms		“I think it would be good if push notifications could come several times a day. Feedback messages arrive during the day, and I want to enter them all at once in the evening, but sometimes I forget and it is already past midnight.”

## Discussion

### Principal Findings

We found that the “My HeartHELP” app may be feasible and acceptable for individuals at risk of cardiovascular disease to be informed of their cardiovascular health, to self-monitor their heart-healthy behaviors, and to adhere to heart-healthy behaviors. Moreover, our data revealed that positive changes in heart-healthy behaviors may be achieved using the “My HeartHELP” app when included as a strategy for heart-healthy behavioral lifestyle interventions.

The “My HeartHELP” app met the criteria of feasibility and acceptability for the validity test of a mobile app. The vast majority of participants (95.6%) completed daily self-monitoring of their heart-healthy behaviors and accessed the app more than once per day (1.8 times). Moreover, the participants perceived the “My HeartHELP” app as comprehensible, easy to use, beneficial to their health, highly usable, and technically complete. Overall, the participants were satisfied with the My HeartHELP app and agreed to recommend it to others. Meanwhile, monitoring one’s own behavior may be directly linked to adherence to healthy behavior [58,59] through the behavioral actions of participants, such as opening the app and entering their daily behavioral outcome for the items. Logging food in real time on mobile devices appears to encourage individuals to become more aware of their food consumption, which may have supported their efforts to achieve dietary goals and weight loss [60,61]. In this context, the action of accessing the app itself may induce motivation to monitor one’s own behavior, which can lead to self-awareness of one’s own behavior and thus to desirable heart-healthy behavior [61].

Our findings revealed that heart-healthy behaviors changed positively over 4 weeks, although significant changes were not verified for all 6 behaviors. Mobile health interventions have revealed inconsistent results for improving heart-healthy behaviors and have reported positive effects limited to a single behavior, that is, physical activity. In this regard, the “My HeartHELP” app is equipped with behavioral strategies that allow individuals to monitor and practice complex and multiple heart-healthy behaviors using a 1-stop approach. Six heart-healthy behaviors via the app were positively changed over the course of 4 weeks, with the limitation of a short duration. These positive results may be explained by installing behavioral strategies for promoting motivation and self-efficacy based on the IMB model [18], that is, goal setting, self-monitoring, and feedback provision. A systematic review reported that behavioral counseling had a small to moderate effect on behavioral interventions when tailored to advice, goal setting, and written material provision [62]. Therefore, we can determine the feasibility and acceptability of the “My HeartHELP” app as a critical strategy for heart-healthy behavioral lifestyle interventions.

We found that the health benefits scores (behavioral information and performance/feedback) and technical completeness score in the acceptability presented the lowest score (3.6 points each), although the acceptability criteria were met (3.5 points). Linked to this finding, qualitative data from the focus group revealed that the participants had requested an additional alarm that could remind them to practice heart-healthy behaviors. Moreover, direct interaction with experts is necessary beyond self-monitoring and the performance interface of healthy behavioral performance [63]. In the future, feedback text messaging in which experts can provide sympathy for and support individual behavioral performance is needed to initiate intervention studies. Finally, the participants mentioned that the information messages were perceived as not individualized to an individual’s lifestyle and situation. This finding indicates that the use of mHealth alone may have limitations in providing information tailored to an individual’s situation and that face-to-face education is needed to provide information tailored to specific individual needs.

### Limitations

This study outlined a few limitations. First, the generalizability of the results to ethnic groups other than Koreans is limited. In addition, to apply it to more people, the app developed in Korean needs to be translated and developed in other languages for linguistic inclusiveness. Second, because it is based on the diet of Koreans, in terms of monitoring eating behavior, its scope of use may be limited. However, because the behavioral strategies of “My HeartHELP” are key skills for behavioral change, the application of “My HeartHELP” to other languages may be necessary. Third, although “My HeartHELP” establishes behavioral goal setting based on standard criteria for adults [38-43,49,51], future studies should further incorporate tailored goal-setting strategies that promote individuals’ autonomy, as emphasized in self-determination theory. Enhancing autonomy in goal setting may foster intrinsic motivation, which is essential for sustaining long-term behavior change [29,64]. Fourth, the 4-week follow-up period may have been too short to capture sustained behavior changes, highlighting the need for longer validation studies. Finally, the use of focus group discussions instead of anonymous methods may introduce social desirability bias, as participants may adjust their feedback based on group dynamics rather than expressing independent perspectives [65].

### Conclusions

“My HeartHELP,” which includes information, self-monitoring, and real-time feedback messaging strategies that promote the practice of complex heart-healthy behaviors, could be feasible and acceptable as an app for promoting cardiovascular health and could contribute to achieving optimal heart health for populations at risk for cardiovascular disease in the community. Practically, community settings, unlike hospital settings, present challenges and limitations in providing intensive professional care for comprehensive heart-healthy lifestyle modification. Therefore, the utilization of the “My HeartHELP” app in health promotion programs aimed at cardiovascular disease prevention at community settings may offer a potential way to solve the limitations.

## Supplementary material

10.2196/66108 Multimedia Appendix 1 Feasibility of the “My HeartHELP” app: Record of self-monitoring for 11 behavioral components of the 6 heart-healthy lifestyle behaviors. A total of 29 participants at risk for cardiovascular disease residing in Seoul, South Korea, took part in the study. The feasibility of the self-monitoring record was assessed based on participant adherence, which was measured by calculating the percentage of participants who adhered to at least 75% of daily self-monitoring completion for each heart-healthy behavior over 4 weeks.

10.2196/66108 Checklist 1 Good reporting of a mixed-methods study (GRAMMS) checklist.

## Abbreviations

IMB: Information-Motivation-Behavioral Skills
