# Supplementary material for: Evaluating Feasibility and Acceptability of the “My HeartHELP” Mobile App for Promoting Heart-Healthy Lifestyle Behaviors: Mixed Methods Study
Source: JMIR Form Res. 2025 May 2;9:e66108. doi: 10.2196/66108 (PMC12064136; doi:10.2196/66108)
Supplement: Multimedia Appendix 1 [file formative-v9-e66108-s001.pptx]

## Slide 1
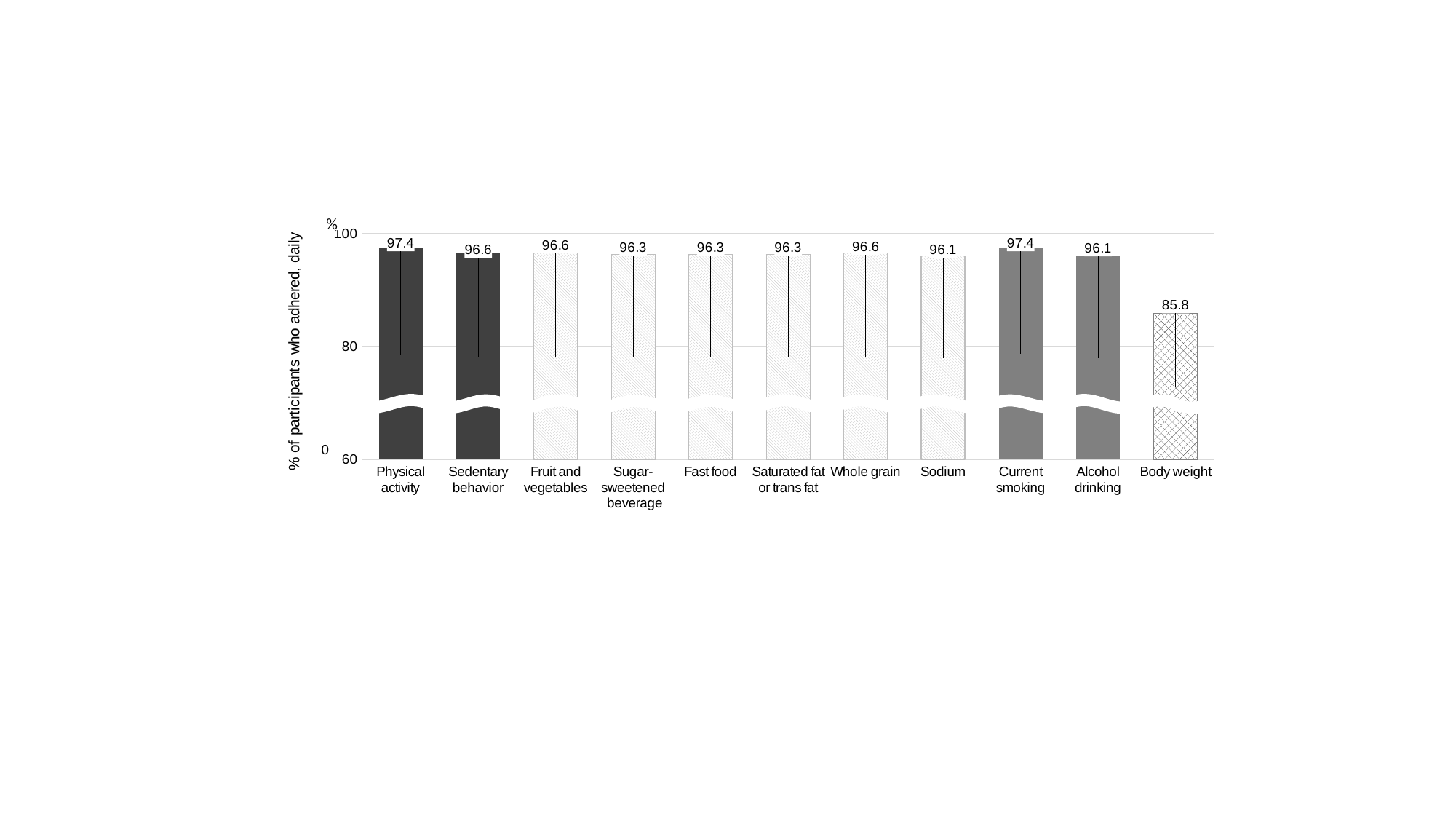

### Chart
| Category | |
|---|---|
| Physical activity | 97.41379310344827 |
| Sedentary behavior | 96.55172413793103 |
| Fruit and vegetables | 96.55172413793103 |
| Sugar-
sweetened
 beverage | 96.30541871921181 |
| Fast food | 96.30541871921181 |
| Saturated fat
or trans fat | 96.30541871921181 |
| Whole grain | 96.55172413793103 |
| Sodium | 96.05911330049261 |
| Current smoking | 97.43589743589743 |
| Alcohol drinking | 96.1038961038961 |
| Body weight | 85.83743842364532 |%
0
