# Supplementary material for: Evaluating Feasibility and Acceptability of the “My HeartHELP” Mobile App for Promoting Heart-Healthy Lifestyle Behaviors: Mixed Methods Study
Source: JMIR Form Res. 2025 May 2;9:e66108. doi: 10.2196/66108 (PMC12064136; doi:10.2196/66108)
Supplement: Checklist 1 [file formative-v9-e66108-s002.docx]

Appendix 1: Good reporting of a mixed-methods study (GRAMMS) checklist

| Guideline | Section: page information |
| --- | --- |
| 1. Describe the justification for using a mixed methods approach to the research question | Methods: p.4 |
| 2. Describe the design in terms of the purpose, priority and sequence of methods | Methods: p.4  P 7-8 |
| 3. Describe each method in terms of sampling, data collection and analysis | [Sampling] Methods: p.4-5  [Data collection] Methods: p.8  [Analysis] Methods: p.10 |
| 4. Describe where integration has occurred, how it has occurred and who has participated in it | Methods: p.4-5 and p.9-10  Results: p.13-14 (Tables 4-5). |
| 5. Describe any limitation of one method associated with the presence of the other method | Methods: p.9  Discussion: p.16-17 |
| 6. Describe any insights gained from mixing or integrating methods | Discussion: p.16-17 |

O'Cathain A, Murphy E, Nicholl J. The quality of mixed methods studies in health services research. J Health Serv Res Policy. 2008;13: 92-98.
